# Clarifying the origin of Houzao

**DOI:** 10.1186/s13020-018-0182-0

**Published:** 2018-05-03

**Authors:** Zhongzhen Zhao, Eric Brand, Hiu Yee Kwan, Quanbin Han, Mengjia Zhou

**Affiliations:** School of Chinese Medicine, Hong Kong Baptist University, Hong Kong, China

**Keywords:** Houzao (bezoar), Goat bezoar, Indian goat, Caecum, Concretion, *Acacia nilotica* (L.) Delile

## Abstract

**Background:**

Houzao (bezoar) is a valuable imported Chinese medicine that is commonly used as a pediatric medicine to transform phlegm. There are mainly two types of Houzao, “Southeast Asian Houzao” and “Indian Houzao”. “Indian Houzao” is the dominant commercial product accounts for over 95% of the actual utilization in the market. However, its origin, formation, composition, efficacy and pharmacology remain unclear. Therefore, we have conducted on-site investigation to clarify the origin of Indian Houzao. We have dissected one male and one female domestic Indian goats in the pastoral areas of Telangana province in south-central India. Our results show:

1. Indian Houzao originates from Indian goats rather than from macaques; it comes from goats and not sheep, and is not limited to female goats.
2. The exact location of the bezoar is in the caecum and not stomach or intestines.
3. Acacia seeds serve as the primer to induce the formation of bezoar in the caecum.
4. The formation and development of the bezoar are closely related to the special local ecosystem and food chain. These goats eat the shoots of *Acacia nilotica*, and also other local plants in the families of Euphorbiaceae, Rutaceae, Combretaceae, etc.
5. It takes around 120 days for the bezoar to be fully developed inside the goat. Many goats are slaughtered in the Indian festival Dusserah from October to December.

Indian Houzao is the bezoar from the caecum of Indian goats, formed in response to pathological stimulation, and is the dominant commercial form of “Houzao” on the market. It has been used historically. It has natural supply source. Producers can guarantee a sustainable supply of the bezoars for the market. The usage of bezoar as medicine is also acceptable from the perspective of animal protection. Many patients and people in the Chinese medicine field do not know Indian Houzao comes from Indian goats but from other unsustainable animal sources, which has a negative influence on its actual use and scientific research potential.

**Conclusion:**

Our study has clarified the origin of Indian Houzao, which can help to further develop Indian Houzao for the treatment of diseases.

## Background

### Application

Houzao (bezoar) is an imported and valuable pediatric Chinese medicine used to transform phlegm [1]. The 《飲片新參》 (*Yinpian xin can*) [2] of The National TCM Pharmacopoeia Prescription Collection [3] has records of different Houzao-containing Chinese medicine formulations collected from different areas, including Shanghai, Nanjing and Hangzhou. Since 1997, China has listed “Houzao San” (“Houzao Powder”) as an essential Chinese medicine for emergency medicine [4]. Clinically, Houzao San is mainly used for the treatment of fever, bronchitis, pneumonia, asthma and patients with symptoms of interior heat [5–13]. At present, there are 11 manufacturers producing Houzao-related products in mainland China. In Hong Kong, more than a hundred manufacturers have registered Houzao-related products, two of which have their products sold in the mainland Chinese market.

### Documentation

Houzao is described in the Chinese Materia Medica (Zhong Hua Ben Cao) as the gastrointestinal concretions of *Macaca mulatta* Zimmermann or *Macaca speciosa* F. Cuvier in the Family Cercopithecidae. The concretion can be collected year-round from the gastrointestinal tract after the animals are killed. The concretion is dried in ventilated place. Production regions include India, the Malay Peninsula and the Malay Archipelago [14].

The earliest record of Houzao dates back to 1930, in one of the Chen Renshan’s writings named *Yaowu Chuchan Bian* (“Analysis of the Production Regions of Medicinals”). This text clarified the origins of Houzao, noting that a stone is developed from the essence of the animal in the stomach, liver and gallbladder, accumulating over years in old monkeys that have long consumed wild fruits. The shape of Houzao is similar to jujubes and it is similar to the bezoar of a cow or a dog, and therefore has similar therapeutic effects [15]. Since then, many academic writings in China describe Houzao as the gastrointestinal bezoar of macaques in the family Cercopithecidae; however, the historical origin of Houzao is still controversial. Indeed, Chen Renshan already questioned the origin of Houzao. Chen mentioned that “the local people from its production regions in the Malay Archipelago refer to it as a goat intestine stone, there must a reason for this.” An early study in 1979 by Zhang [16] and others also pointed out that the stones of macaques and goats appear morphologically similar but differ in the features of their cross-section, allowing for differentiation.

To clarify the identity of Houzao, the National Pharmaceutical Administration organized a site visit to India in 1985. The investigation concluded that Houzao is a bezoar formed in the gastrointestinal tract of female goats [14]. Subsequent publications frequently repeated this conclusion [17–20]. Yu [21] and others used X-rays to analyze the diffraction patterns of Malaysian Houzao and Indian Houzao, and the results indicated that they have different compositions. In 1984, in issue 5 of the Journal of the Chinese Herbal Medicine, Qiu [22] identified the difference between Malaysian Houzao and Indian Houzao. He reported that Houzao was derived from processed or semi-processed animal bezoars. Xue and Yi [23] found that Houzao contains bile but is not a gallstone. They hypothesized that Houzao consists of seeds or other debris that serves as the core, and is surrounded by layers of a mixture of bile in goats, monkeys or other animals; over time the bezoar forms in the stomach or gallbladder of a goat or monkey, then it is removed for grinding and further processing. Sun [24] also suggests that Houzao is not the bezoar of a monkey, but is a kind of gastrointestinal phosphate-carbonate concretion like the bezoar of a horse or dog, which can be cultivated and produced like the bezoar of cow.

### Market research

To investigate the origin of Houzao, we paid a visit to a senior in the field of traditional Chinese medicine in Hong Kong, Mr. Lee Chun Hung. Mr. Lee is over 80 years old, and has been selling valuable Chinese medicines in Hong Kong for more than 60 years. His shop has a collection of many precious Chinese medicines, including different types of Houzao. Mr. Lee describes Houzao as an imported medicinal. There are two kinds of Houzao with two product specifications: the first kind is the “Houzao” that comes from Malaysia, while the other kind is called “Indian Houzao” that comes from India (it is recognized as an intestinal bezoar from goats). Historically, Houzao did not refer to a bezoar from the gastrointestinal tract, but rather referred to a bezoar formed in the cheek pouch of macaques in Southeast Asia. Macaques store food in their cheek pouches and later consume this reserve slowly. If there are pebbles or twigs that cannot be digested, the macaque will secrete a protective membrane around the debris to avoid inflammation or damage. The secretion will grow thicker and gradually form into a bezoar. However, the annual supply of such bezoars from macaques is only around a dozen kilograms worldwide, and is occasionally mixed with bezoars from other monkeys or apes. The origin of such bezoars is unclear and cannot be traced, with an unstable supply that risks decline and extinction. Mr. Lee stated that goat bezoars where originally produced in Iran, but were later sourced from India. Every year, about 200–400 kg of Houzao (goat bezoars) are available on the market. In addition to being used as single product, they are often used in compound Chinese medicine products. Within the industry, the goat bezoars are also referred to as *yang chang zao* (羊腸棗, “goat intestine jujubes”). He believes that goat bezoars have been traded in China for at least 200–300 years.

We also contacted the two Hong Kong manufacturers of Houzao-related products, “Po Wo Tong Pharmaceutical Limited” and “Eu Yan Sang”. These companies confirmed that 100% of the Houzao they use for their products is the goat bezoar form (*yang chang zao*羊腸棗).

In the March of 2018, we have paid a visit to Mr. Jin Shi-yuan, a master Chinese medicine practitioner in Beijing. Mr. Jin has been engaged in the Chinese medicine industry for nearly 80 years. He describes Houzao as similar to the gallstone of a cow; both are traditionally well-known as imported and valuable Chinese medicines. While circumstances did not allow him to have the chance to conduct a site visit to investigate the origins of Houzao, he is familiar with it. Currently, most local manufacturers are mainly in the south of China, such as Shanghai, Hangzhou, and Nanjing. In his memory, the Houzao he observed were similar, with a flat and oval shape and shiny blue color; authentic products make a sound when shaken together.

### Summary

In summary, many professionals in the TCM industry suspected that Houzao is not derived from a single kind of animal. It is also acknowledged that Indian Houzao comes from a goat rather than a monkey; however, it is not known if other goat breeds can also produce such bezoars. Some have speculated that a portion of the Houzao on the market is artificially produced in goats. However, thus far no professionals in the Chinese medicine field have visited India to conduct on-site investigation and witness the production and collection of Houzao; instead, they primarily receive information through the descriptions of local traders. To date, since none of the published data describes the collection of the bezoar from macaques or goats based on eyewitness reports, the speculative ideas referenced above lack a concrete answer.

Accordingly, in the mid-January in 2018, we visited south-central India to investigate the origin of the Houzao that dominates the market, to explore where it comes from, how it is formed, how the food chain affects its formation, and how it is collected.

## The field investigation

### The environment

Our on-site investigation was conducted in Telangana province in south-central India, which is a mixed farming and pastoral area. It produces cotton, corn, rice and fruits, and has both dry and wet seasons.

We were led by Mr. Aijaz Mahboob Khan (A. M. Khan), who is specialized in the local Houzao business. After four hours of driving from Hyderabad, we reached Karimnagar and Warangal on the first day; we went to Nizamabad on the second day. We visited two shepherd families in the two villages and dissected two domestic Indian goats on-site. The first goat we dissected was a male goat, the second one was a female goat. Mr. Huan Yiping and Mr. Chai Lin recorded the whole dissection process. We also collected some plant specimens, which are now kept in the Chinese Medicines Center of the Bank of China and School of Chinese Medicine in Hong Kong Baptist University (Nos. ZZZ2018-India001 to 006).

### Formation of the bezoar

The area is home to many goats (*Capra aegagrus hircus*), which have horns and dark brown to black bodies. These goats usually weigh between 10 and 20 kg. They have a long head, short neck and large ears. The males and females have a pair of horns at the forehead, with larger horns on the males. The jaws of the males have long whiskers. They have 4 thin limbs and a short tail, and their entire body is covered by coarse, straight, short hair. The hair ranges in color from black, gray, or brown, sometimes with other combinations. In general, female goats can become pregnant twice a year, each time giving birth to 2–4 kids; it takes 6 months for a kid to grow into an adult.

The Indian black goats live as a herd. They are strong foragers and eat many varieties of grass, trees and leaves. In April, after the fruits of the tree “Tumma chettu” (*Acacia nilotica*) mature, the local people tap the branches with sticks to harvest the fruits and collect the seeds. Since the seeds are bitter, they are soaked in salt water before being fed to the goats.

Experienced shepherds will know if the goats have the potential to accumulate the seeds by touching the goat’s abdomen; goats that do not tend to accumulate the seeds will be taken to a normal pasture and will be raised for meat; the goats that tend to accumulate the seeds will be taken to the forest to graze on specific plants. They are transported deep into the mountains to be raised in remote camps in the forest, where they are fed specific plants to facilitate the formation of the bezoars. The grazing period starts from the wet season in June, when the local vegetation flourishes. In the dry season in November, the goats are slaughtered for meat and the bezoars are collected. It takes approximately 120 days for the bezoar to be fully formed inside the goat after feeding on the *Acacia* seeds.

In the Chinese literature, it is incorrectly stated that only female goats can produce bezoars. Indeed, both male and female goats can produce bezoars.

### The food chain of the *Capra aegagrus hircus*

The food chain is closely associated with the formation of bezoars in goats.

**Fig. 1:**
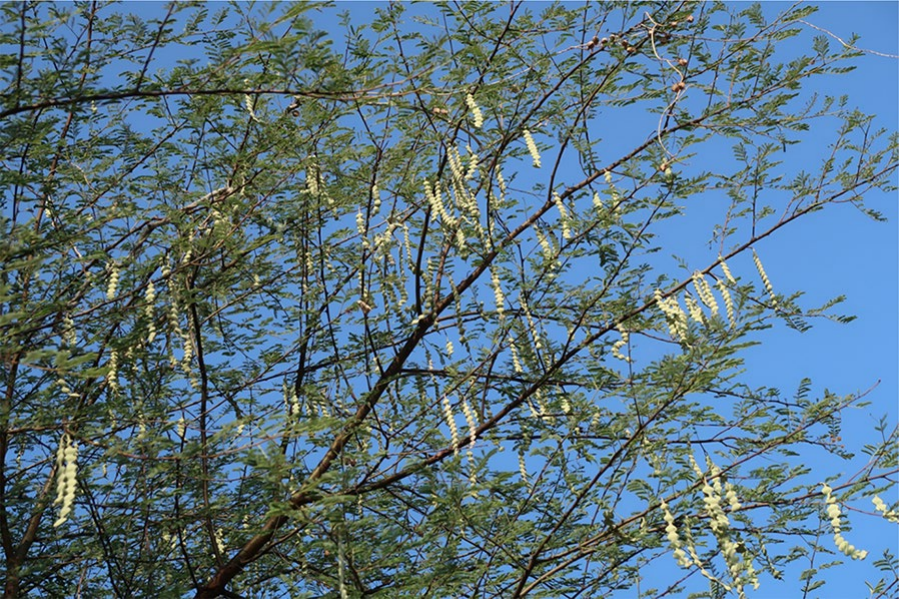
*Acacia nilotica* (L.) Delile, a leguminous acacia plant

Accompanied by local shepherds, we observed trees that the local people call “Tumma chettu” (babul trees). The trees are three to five meters tall, with beige-colored bark and hairy shoots. They have 2–11 pairs of pinnulae, 7–25 pairs of leaflets, and are oblong with soft hairs. The pods are strip-shaped, straight or curved, and can be up to 20 cm long and 1–2 cm wide. Their seeds are constricted and tomentose, covered by velvety hairs, and are not fissured. The tree was later identified as *Acacia nilotica* (L.) Delile, a leguminous acacia plant (Fig. 1). The Acacia pods generally mature in April. We arrived there in mid-January, and the pods were still tender with a sticky surface. They appear similar to the fruit of the Japanese pagoda tree (*Sophprae fructus*). The seeds inside the pods taste bitter, and turn brown when they are mature. The Acacia wood is hard, reddish brown, water tolerant and resists termites. It can be used for making furniture and farm tools. Both the bark and pods contain tannins, which are used for tanning leather in the local leather industry. The branches and young leaves are used to feed cattle and goats. The gum from these trees is a source for the product gum arabic, which is primarily used in the printing and dyeing industry.

*Acacia nilotica* (L.) Delile originated in Africa and spread to Arabia, Afghanistan and India. It is now cultivated worldwide. In China, it is cultivated in Hainan, Yunnan and other regions.

In the field, we also observed leguminous relatives of *Acacia nilotica,* such as *Albizia procera* (Roxb.) Benth, and many other species of plants that the goats eat while foraging, including:

- *Anogeissus latifolia*, Combretaceae;
- *Cleistanthus collinum*, Euphorbiaceae,
- *Bridela montana*, Euphorbiaceae,
- *Chloroxylon swietenia*, Rutaceae,
- *Tamarindus indica*, Fabaceae.

These plants flourish from the beginning of the wet season in early June until November, and provide ample food for the goats during the grazing period. The bezoar is thus produced by feeding specific plants to the goats.

### The collection of the goat bezoar (羊腸棗)

**Fig. 2:**
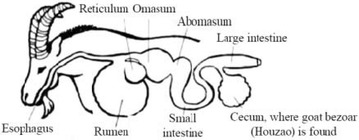
The digestive system of the goat (prepared by Lam Yin Ching)

**Fig. 3:**
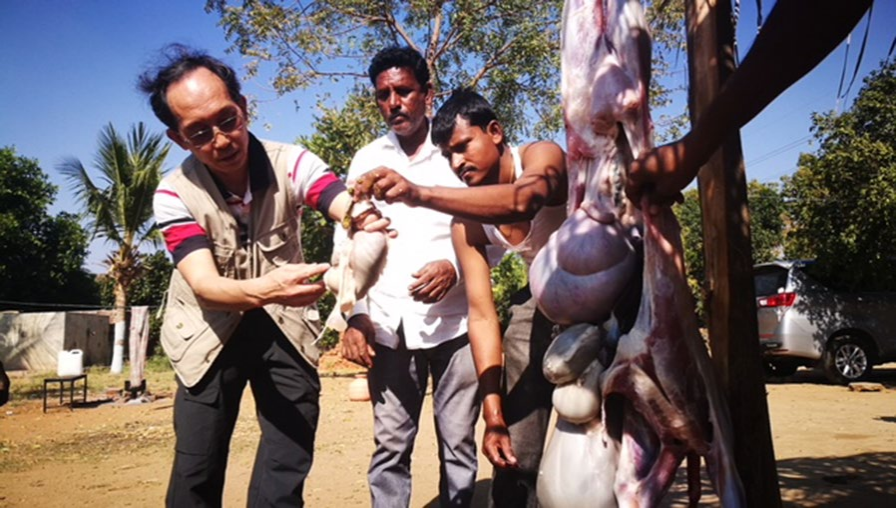
Collection of goat bezoar from the Indian goat *Capra aegagrus hircus*

Goats are herbivores; their digestive system is illustrated in Fig. 2. Together with the local shepherds, we dissected two domestic Indian goats on-site, one is male goat, the other is female goat (Fig. 3). The bezoar is formed in the caecum, which the local people refer to as “cirdan”. Experienced farmers can feel the bezoar in the abdomen of the goat to non-invasively monitor its development.

After they fall into the caecum of the goats, the acacia seeds are not excreted and serve as the “primer” to induce the secretion of anti-inflammatory substances to protect the goat from irritation by the seed. Since the seeds are rich in tannins, anti-inflammatory substances coagulate with the proteins to form a natural protective layer on the surface.

**Fig. 4:**
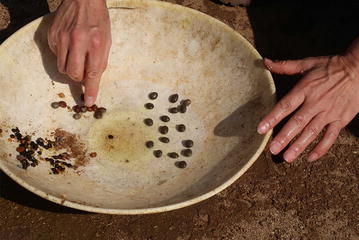
Bezoars obtained in the goat we dissected in the on-site visit

**Fig. 5:**
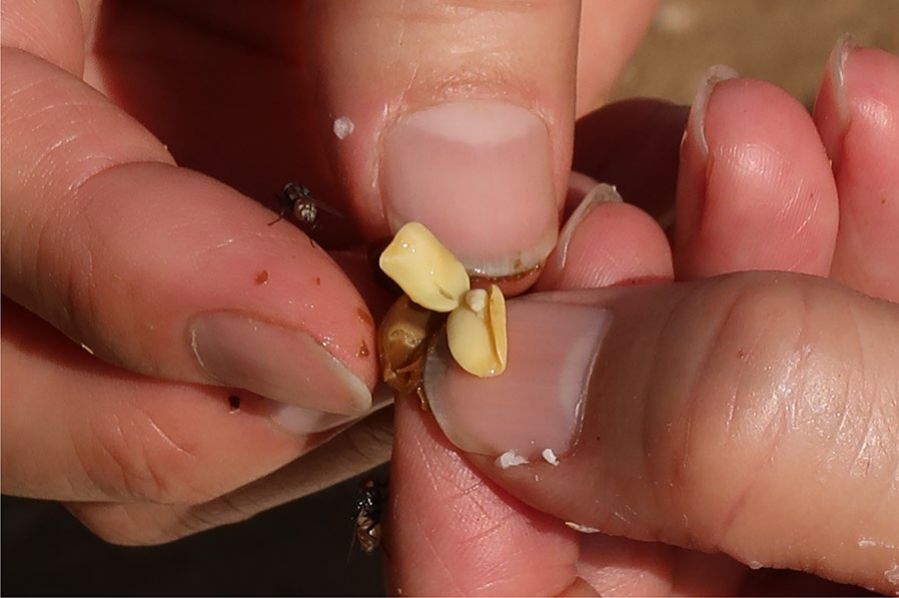
The two bean-shape cotyledons of the acacia seed in the bezoars which we obtained in the goat

After rinsing the yellowish-green semi-liquid material collected from the caecum, the dark brown, shiny bezoars can be seen. In the goat that we dissected, a total of 17 bezoars were obtained (Fig. 4). After removing the outer gray layer of the bezoar that covers the seed coat, we could observe the two bean-shape cotyledons of the acacia seed (Fig. 5). In the same caecum sac, we also observed some pebbles and a few black beans. Interestingly, these beans lacked a protective outer layer, suggesting that the bezoar is only formed on the surface of the acacia seeds.

### Collection

The Dusserah legend comes from the ancient epic Ramayana of India. After being exiled by the King for 14 years, Prince Ram eventually rescued his wife, Sita, from Devil’s Island and returned to her hometown, the whole country welcomed the goddess Lakshmi. Local people celebrate this traditional event by setting up thousands of goat feasts. According to the traditional Indian calendar, Dusserah is usually between October and December each year. At this festival, many goats are slaughtered and the meat is consumed.

Each goat can produce around 5–7 g of bezoars (5–10 pieces of bezoars). Usually, there are 2500–3000 bezoars in 1 kg. Thus, it takes approximately 175 selected goats to produce 1 kg of bezoars. A 1-year-old goat usually weighs 10–15 kg; a whole goat can only be sold for approximately $300 Hong Kong Dollars (~$38 US dollars) for its meat, and its skin is not valuable. However, if there are bezoars in the goat, the shepherd can earn more from the bezoars than from the meat. Therefore, herding goats that produce bezoars is a good way of earning income for the local people.

## Identification of the medicinal materials

### Identification and characterization of goat bezoar (*yangchangzao*, 羊腸棗) (Indian Houzao) (bezoar from the caecum of Indian goat *Capra aegagrus hircus*)

**Fig. 6:**
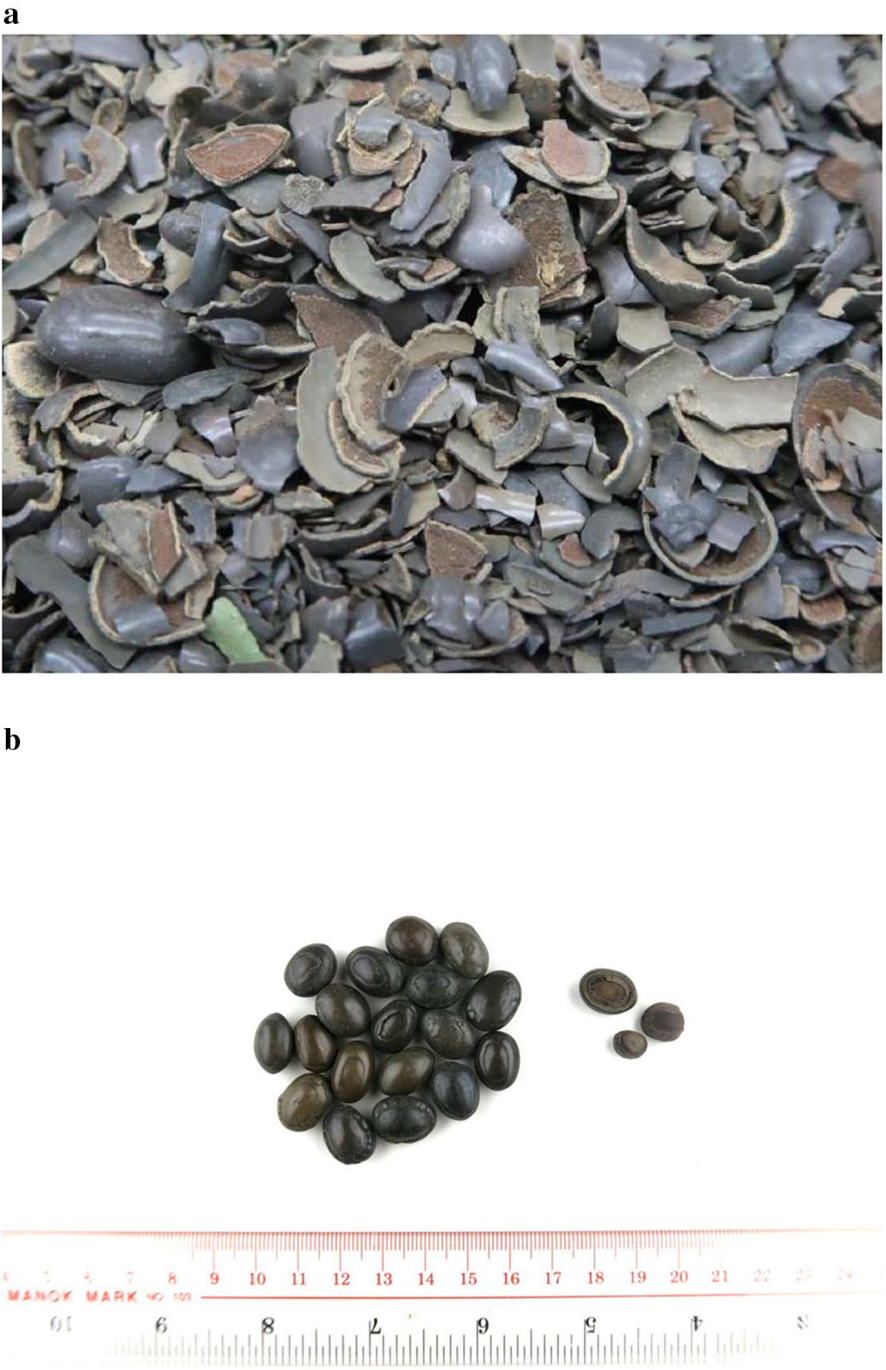
The goat bezoar (yangchang zao, 羊腸棗) (Indian Houzao) (bezoar from the caecum of Indian goat *Capra aegagrus hircus*). **a** The commercial product. **b** The commercial product with a scale bar

They are mostly oval or oblong in shape; flattened and small. They are similar in size, and are approximately 1.5–2.0 cm in length and 1.0–1.5 cm in diameter. They have a dark green or dark brown surface, are smooth and shiny, have a concentric ring near the edge, and occasionally have verrucous protrusions at the edges. They are light in weight, hard but brittle and fragile. The cross-section is about 1–2 mm in thickness, with obvious layers, and is easily peeled off. The inner surface is a little rough and dull. The center has a big hole embedded with a long oval seed. The seed (acacia seed) is dark brown on the surface, smooth and slightly shiny. The seed also has a concentric ring near the edge, which is consistent with the appearance of the medicinal material, with a width of 0.6–1.0 mm. It is also light in weight, hard and brittle, and has a thick outer skin. After using water to soak the seed and then peel off the skin, two cotyledons can be seen. The bezoar has a slight fragrance, and is slightly bitter and astringent, with a gritty texture when chewed (Fig. 6).

### Identification and characterization of Southeast Asian *Houzao* (bezoar from the cheek pouch of macaques)

**Fig. 7:**
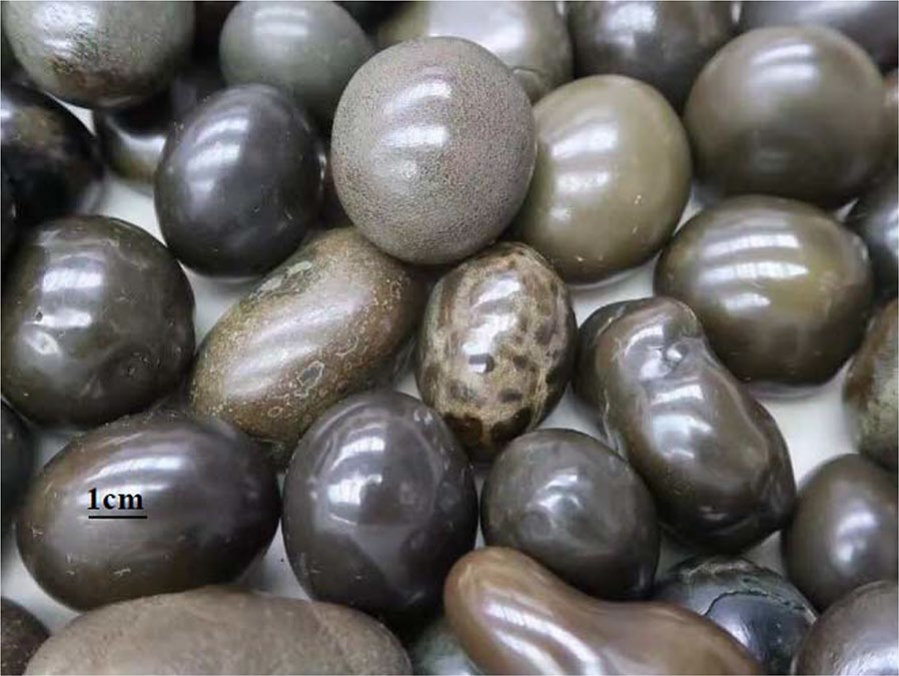
Southeast Asian Houzao (bezoar from the cheek pouch of macaques), the commercial products

They are oval and vary in size, and can be as big as an egg (3.0–4.0 cm in length, 1.8–2.2 cm in diameter). More commonly they are the size of a lotus seed and can be as small as a soybean. They have a bronze or greenish black surface, and are smooth and shiny; some have verrucous protrusions. The center is solid and they are hard but brittle and fragile. The cross-section appears grayish-yellow and rough. They resemble a cow gallstone with layers, and have a bitter taste and gritty texture (Fig. 7).

## Discussion and conclusion

Houzao (bezoar) is a valuable, imported Chinese medicine that is commonly used as a pediatric medicine to transform phlegm. However, its origin, formation, composition, efficacy and pharmacology remain unclear. In this study, with a focus on “Indian Houzao” (the dominant commercial product), we have conducted an on-site investigation and dissected two domestic Indian goats in two villages in Telangana province in south-central India. Our results show:

1. The origin and fact: The Indian Houzao available in the market originates from Indian goats but not from macaques or sheep, and is not limited to female goats. These are domestic goats but not wild goats. The exact location of the bezoar is in the caecum instead of the stomach or large intestine tract. Acacia seeds serve as the primer to induce the formation of the bezoar in the caecum, and the formation and development of the bezoar are closely related to the local habitat and food chain. These goats eat the shoots of *Acacia nilotica*, and also other local plants in the families of Euphorbiaceae, Rutaceae, Combretaceae, etc. It takes around 120 days for the bezoar to be fully developed inside the goat.
2. Confirmation of the Chinese nomenclature: We propose to use the Chinese name *Yangchang zao* (羊腸棗) (also known as Indian *Houzao*), which can clearly indicate that it is a bezoar formed in the caecum of Indian goat (*Capra aegagrus hircus*), and the bezoar is formed with the acacia seed at its core. Firstly, the name *Yangchang zao* (羊腸棗) is scientifically based. Secondly, this name has already been recorded by professionals, and has the same meaning as the name that is being used in the country of origin. Furthermore, although it is more accurate to name it as “bezoar from the caecum of a goat”, the proposed name *Yangchang zao* (羊腸棗) will easily accepted by the pharmaceutical industry and the general public. In short, the name *Yangchang zao* (羊腸棗) does not only inherit the history of this traditional medicine, but is also scientifically based.
3. Bezoar as a detoxification ingredient has been mentioned in the worldwide best-selling novel “Harry Potter and the Half-Blood Prince”. The book says, “bezoar is an antidote to all poisons.” [25] Goat bezoar has a long history in European culture as well.
  Goats are successfully raised worldwide, and goat bezoar is sustainable from the perspective of animal protection, so *yangchang zao* (羊腸棗) has the potential to be a widely accepted Chinese medicinal material (CMM) by the general public.
4. There is a rich variety of CMMs, and each has its own history. The famous physician Li Shizhen stated that different herbal medicines were popular between ancient times and the contemporary period he lived in. Historically, some CMMs have been continued while others have been changed.
  There are two main criteria that are necessary for a CMM to become a principal or authentic source: (i) reliable therapeutic efficacy and (ii) a rich source of supply [26]. Goat bezoar (*yangchang zao*羊腸棗) (Indian Hauzao) has been used historically and it has a stable supply source. In south-central India, there are suitable plants and a suitable environment for grazing by goats. Furthermore, sophisticated local cultivation techniques guarantee a sustainable supply of the bezoars for the market. Therefore, goat bezoar (*yangchang zao*羊腸棗) deserves an in-depth investigation.
5. The Houzao available in the market is commonly used in scientific research [27]. However, we should take into consideration that the biological compositions of the seed kernel and the bezoar shell of the kernel are different. Pharmacological and clinical studies mainly focus on the compound Chinese proprietary medicine “*Houzao san*”. The reported pharmacology and therapeutic efficacy of the multicomponent “*Houzao san*” cannot represent the pharmacology and therapeutic efficacy of the single bezoar on its own (*yangchang zao* 羊腸棗). Therefore, after clarifying the origin, we should conduct chemical, pharmacological and clinical research on goat bezoar (*yangchang zao*羊腸棗, Indian *Houzao*), which can provide solid scientific data for future development, production and application.
6. Ancient Chinese medicine has always incorporated foreign medicinal products. Clarifying the origin of medicinals is necessary to ensure that the public has access to authentic and high-quality medicines.
